# Healthy Vision Month — May 2013

**Published:** 2013-04-26

The May 2013 theme for Healthy Vision Month is “Healthy Vision: Make It Last a Lifetime.” CDC’s Vision Health Initiative joins with the National Institutes of Health’s National Eye Institute in encouraging everyone to make vision and eye health a lifetime priority.

In 2010, approximately 4 million persons in the United States aged ≥40 years had vision impairment (including low vision and blindness); by 2050, this number is projected to reach 13 million (1). Vision impairment is associated with inability to perform daily activities such as reading, driving a car, and preparing meals. Vision impairment also is associated with an increased risk for falls, fall-related injuries, depression, and reduced overall health (2–4). Millions of persons in the United States have undetected vision problems and eye diseases. Vision disorders are the seventh most common chronic condition for persons aged ≥65 years, the ninth most common for those aged 50–64 years, and the third most common for those aged ≤17 years (5,6).

Early detection, timely treatment, and the use of proper eye safety practices can prevent or delay vision impairment. The American Optometric Association and the American Academy of Ophthalmology recommend a regular, comprehensive dilated eye examination to potentially detect and treat vision problems early. Additional information about activities that promote prevention, early detection, and treatment of eye diseases leading to vision impairment is available at http://www.cdc.gov/visionhealth and http://www.nei.nih.gov/healthyeyes.
